# Proper Generalized Decomposition for Parametric Study and Material Distribution Design of Multi-Directional Functionally Graded Plates Based on 3D Elasticity Solution

**DOI:** 10.3390/ma14216660

**Published:** 2021-11-04

**Authors:** Mohammad-Javad Kazemzadeh-Parsi, Francisco Chinesta, Amine Ammar

**Affiliations:** 1 Department of Mechanical Engineering, Shiraz Branch, Islamic Azad University, Shiraz 71987-74731, Iran; 2 LAMPA, Arts et Metiers Institute of Technology, 49035 Angers, France; amine.ammar@ensam.eu; 3 PIMM Lab & ESI Chair, Arts et Metiers Institute of Technology, 75013 Paris, France; francisco.chinesta@ensam.eu

**Keywords:** proper generalized decomposition, separated representation, functionally graded material, material distribution design, plate bending

## Abstract

The use of mesh-based numerical methods for a 3D elasticity solution of thick plates involves high computational costs. This particularly limits parametric studies and material distribution design problems because they need a large number of independent simulations to evaluate the effects of material distribution and optimization. In this context, in the current work, the Proper Generalized Decomposition (PGD) technique is adopted to overcome this difficulty and solve the 3D elasticity problems in a high-dimensional parametric space. PGD is an a priori model order reduction technique that reduces the solution of 3D partial differential equations into a set of 1D ordinary differential equations, which can be solved easily. Moreover, PGD makes it possible to perform parametric solutions in a unified and efficient manner. In the present work, some examples of a parametric elasticity solution and material distribution design of multi-directional FGM composite thick plates are presented after some validation case studies to show the applicability of PGD in such problems.

## 1. Introduction

Shear deformations are important in the flexural behavior of thick plates, especially when they consist of composite or Functionally Graded Materials (FGM). Generally, flexural plate theories (classical and higher orders) assume a predefined displacement profile and shear effects over the thickness and, although this is sufficiently accurate in thin plates, their accuracy in thick plates, especially those involving a composite and FGM, have been in debate for a long time. In fact, the perfect approach in such cases is using the 3D elasticity theory to accurately consider all strains. Unfortunately, the use of traditional mesh-based numerical methods for solving plate problems based on 3D elasticity leads to huge computations, which makes parametric studies more difficult. Therefore, the main goal of the current work is to adopt an approach based on the PGD method that makes it possible to deal with 3D elasticity solutions of plate problems, parametric studies and material distribution design problems, while consuming small computational resources.

PGD is an a priori model order reduction technique, which is based on a separated representation of field functions. In other words, field functions are defined as a product of a univariate function, each of them in one coordinate direction of the problems space, $\Omega \in R^{N_{D}}$. The problem space Ω is the geometrical space (x,y,z) extended by the extra problem-specific parametric space $\left(p_{1},p_{2},\ldots \right)$. The PGD technique reduces the solution of an $N_{D}$-dimensional problem defined in Ω to a series of 1D sub-problems. This technique reduces computational costs considerably, and makes it possible to deal with problems in high-dimensional coordinate spaces. Due to these advantages, the application of PGD has been quickly extended in a variety of problems in science and engineering. For a review on different applications of PGD, refer to [1].

For some pioneering works in the context of the application of the PGD technique in plate-like structures, refer to [2,3,4,5]. There have also been some new publications in this field [6,7,8,9,10]. Although PGD is quite efficient in parametric and high-dimensional problems and the parametric analysis/optimization of FGM materials is highly demanding, there are no published works (to the best of the authors’ knowledge) regarding the application of the PGD technique in FGM materials.

Many works have been published on the use of the 3D continuum theory of elasticity for FGM plate problems and, while the majority of them consider uni-directional plates—whose gradation takes places in the thickness direction (for a review, refer to [11,12])—, there are some works considering in-plane gradation (bi-directional); for an example, refer to [13] for a bending analysis of bidirectionally graded isotropic plates using FEM, [14] for a free vibration and buckling analysis of rectangular and skew isotropic plates with in-plane gradation using scaled boundary FEM and [15] for a free vibration analysis of a bidirectionally graded isotropic plate using the Differential Quadrature Method (DQM). There are very few works considering tri-directional plates. For example, in [16], a thermo-elastic analysis of isotropic plates graded in all of the three directions is considered using DQM.

The above-mentioned works on the 3D elasticity solution of FGM plates only consider isotropic materials, whereas there are few works considering orthotropic behaviors. For example, a semi-analytical hybrid DQM approach is used in [17] for a bidirectional FGM orthotropic plate. The extended Kantorovich method is used in [18] to analyze the static deflection of an orthotropic plate considering Levy-type boundary conditions. A combination of the Kantorovich and power series approaches is used in [19] for the elasticity solution of in-plane grading orthotropic FGM plates.

A careful consideration of the above-mentioned works shows that:

- FGM plates with gradation in directions other than the thickness direction are of current research interest, both from a theoretical and industrial point of view.
- In most cases, the Poisson’s ratios are considered constants, or a similar grading function is used for different orthotropic moduli components (e.g., [20]) or directly for the orthotropic stiffness coefficients (e.g., [17]). These non-physical assumptions are performed to simplify the solution procedure, but can represent crude approximations in many cases.
- Shear deformations are important in thick plates and, although high-order plate bending theories are adopted to somehow consider it, the use of the 3D elasticity approach is inevitable. There are only a few of this type of solution, and even fewer for cases of orthotropic FGM plates and rarely for parametric analyses and material distribution design problems.
- There are some analytical or semi-analytical solutions for three-dimensional elasticity plate problems. Although these methods are successful in decreasing computational costs, they are limited in special boundary conditions, loading and material characteristics, and their application in general plate problems is restricted.
- Parametric studies and material distribution design problems are very limited because they need many independent simulations, which leads to high computational costs using numerical solutions of 3D elasticity problems.
- To the best of the authors’ knowledge, PGD, as an advanced model order reduction technique, has not been applied in the elasticity solution of FGM composite plates thus far.

Based on the above observations, the main motivations and objectives of the present work are to consider all of the above-mentioned points and determine new contributions in the field of the analysis of thick FGM plates. More specially, in the current paper:

- Material grading is considered along three physical directions.
- Orthotropic FGM material constants are considered, consistent with real physics, based on the volume fraction of the constitutive materials and using established micromechanical models.
- An analysis is conducted based on the 3D theory of elasticity to consider shearing deformations perfectly.
- All types of boundary conditions are considered without any limitations.
- Parametric studies are performed in an efficient manner in a high-dimensional coordinate space.
- The application of the PGD technique in the parametric study of thick FGM plate problems is introduced for the first time. The material distribution design is then determined using the resulting parametric study.

In the following, after introducing the governing equations, the notion of a separated representation is introduced and used for representing the space distribution of material characteristics and, also, the displacement field. After that, the PGD technique is used to obtain the unknown displacement field functions. To verify the method, some validation examples are presented and the results are compared with available ones. Further examples are also presented to show the applicability of the method in the material distribution design problems.

## 2. Governing Equations and Weak Form

The static equilibrium equations for a 3D continuum domain considering body force $\left(f_{x},f_{y},f_{z}\right)$ are as follows:

(1) $$\begin{array}{c} \sigma_{xx,x}+\sigma_{xy,y}+\sigma_{xz,z}+f_{x}=0\\ \sigma_{xy,x}+\sigma_{yy,y}+\sigma_{yz,z}+f_{y}=0\\ \sigma_{xz,x}+\sigma_{yz,y}+\sigma_{zz,z}+f_{z}=0 \end{array}$$

The linear elastic stress–strain relation is given by:

(2) $${\left[\sigma_{xx},\sigma_{yy},\sigma_{zz},\sigma_{xy},\sigma_{yz},\sigma_{zx}\right]}^{T}=C{\left[\epsilon_{xx},\epsilon_{yy},\epsilon_{zz},\gamma_{xy},\gamma_{yz},\gamma_{zx}\right]}^{T}$$

The elements of the elasticity matrix, C, are different functions of coordinate components *x*, *y* and *z*, as well as the volume fraction, *V*, of different components which constitute the medium and, also, physical properties of the components itself. The details of the calculation of the elements of C are given in references such as [21].

The strain–displacement relations for small deformations ignoring the effects of temperature changes are as follows:

(3) $$\begin{array}{cc} \epsilon_{xx}=u_{x,x},\;\epsilon_{yy} & =u_{y,y},\;\epsilon_{zz}=u_{z,z}\\ \gamma_{xy}=u_{y,x}+u_{x,y},\;\gamma_{yz}= & u_{z,y}+u_{y,z},\;\gamma_{zx}=u_{x,z}+u_{z,x} \end{array}$$

We used the weighted residual method to obtain the integral form of Equation (1), and performed the integration by part; then, by introducing Equations (2) and (3), the weak form of the governing equations and natural boundary conditions would be obtained as follows. The detailed derivations can be found in [21].

(4) $$\begin{array}{c} \int_{\Omega }\left(u_{x,x}^{*}\left(C_{11}u_{x,x}+C_{12}u_{y,y}+C_{13}u_{z,z}\right)+u_{x,y}^{*}C_{44}\left(u_{y,x}+u_{x,y}\right)\right.\\ \left.+u_{x,z}^{*}C_{66}\left(u_{x,z}+u_{z,x}\right)\right)d\Omega =\int_{\Gamma_{N}}u_{x}^{*}t_{x}d\Gamma +\int_{\Omega }u_{x}^{*}f_{x}d\Omega \end{array}$$

(5) $$\begin{array}{c} \int_{\Omega }\left(u_{y,x}^{*}C_{44}\left(u_{y,x}+u_{x,y}\right)+u_{y,y}^{*}\left(C_{12}u_{x,x}+C_{22}u_{y,y}+C_{23}u_{z,z}\right)\right.\\ \left.+u_{y,z}^{*}C_{55}\left(u_{z,y}+u_{y,z}\right)\right)d\Omega =\int_{\Gamma_{N}}u_{y}^{*}t_{y}d\Gamma +\int_{\Omega }u_{y}^{*}f_{y}d\Omega \end{array}$$

(6) $$\begin{array}{c} \int_{\Omega }\left(u_{z,x}^{*}C_{66}\left(u_{x,z}+u_{z,x}\right)+u_{z,y}^{*}C_{55}\left(u_{z,y}+u_{y,z}\right)\right.\\ \left.+u_{z,z}^{*}\left(C_{13}u_{x,x}+C_{23}u_{y,y}+C_{33}u_{z,z}\right)\right)d\Omega =\int_{\Gamma_{N}}u_{z}^{*}t_{z}d\Gamma +\int_{\Omega }u_{z}^{*}f_{z}d\Omega \end{array}$$

In Equations (4)–(6), $u^{*}$ is the first variation of the displacement components and $\left(t_{x},t_{y},t_{z}\right)$ is the traction vector on the natural boundary $\Gamma_{N}$. The left-hand side of Equations (4)–(6) consists of 21 terms, all of which have the same structure. In other words, the three equations given in Equations (4)–(6) could be rewritten as the following generic form:

(7) $$\sum_{a\ldots f\in \{S\}}\int_{\Omega }u_{a,b}^{*}u_{c,d}C_{ef}d\Omega =\int_{\Gamma_{N}}u_{a}^{*}t_{a}d\Gamma +\int_{\Omega }u_{a}^{*}f_{a}d\Omega$$

where the indices a,b,c,d,e and *f* should be selected according to set {S} to generate all terms in Equations (4)–(6). Set {S} is given in Table 1 for each term of these equations.

The elasticity matrix C in Equation (2) consists of nine nonzero elements, $C_{ef}$, for orthotropic materials. In FGM materials, these coefficients are continuous functions of space coordinates. For instance, the space distribution of $C_{ef}\left(x,y,z\right)$ depends on the space variation of the volume fraction of the different material components which constitutes the FGM and, also, their physical characteristics. The most realistic approach to obtain these elements was by using, at first, a micromechanical model to obtain the engineering elastic constants (elastic modules and Poisson ratios) and, then, using the generalized Hook law for orthotropic materials to obtain the space distribution of the elasticity coefficients $C_{ef}\left(x,y,z\right)$ (see [21]). This approach was used in the current work.

## 3. Separated Approximate Representation (SAR)

Consider $g(x,y,z,p_{1},p_{2},\ldots )$ as a generic field function defined in the problem space, $\Omega \in R^{N_{D}}$. The field function g(x), where $x=(x,y,z,p_{1},p_{2},\ldots )$, may be an unknown field such as displacement component $u_{a}$ or a known field such as body force component $f_{a}$ or the elements of the elasticity matrix $C_{ef}$. Neverhteless, it is generally possible to find a separated approximate representation (SAR), $g^{h}\left(x\right)$, for the generic field function g(x) as follows:

(8) $$g\left(x\right)\approx g^{h}\left(x\right)=\sum_{i=1}^{N_{G}}X_{i}\left(x\right)Y_{i}\left(y\right)Z_{i}\left(z\right)P_{1i}\left(p_{1}\right)P_{2i}\left(p_{2}\right)\ldots$$

where $X_{i}\left(x\right)$, $Y_{i}\left(y\right)$, $Z_{i}\left(z\right)$, $P_{1i}\left(p_{1}\right)$, … are modes in *x*, *y*, *z*, $p_{1}$, … directions, respectively. Equation (8) defines a separated approximate representation of field function g(x). In other words, the approximated field $g^{h}\left(x,y,z,p_{1},\ldots \right)$ was constructed by a superposition of different functions, each of them consisting of a product of univariate functions in different directions of the problem space. Hereafter, just for simplicity, we dropped the superscript *h*, but we knew that the above separated representation was an approximation because the number of modes, $N_{G}$, was considered finite.

Modes $X_{i}\left(x\right)$, $Y_{i}\left(y\right)$, $Z_{i}\left(z\right)$, $P_{1i}\left(p_{1}\right)$, … could be written in terms of a set of approximation functions and corresponding coefficients as used in traditional function approximation approaches as follows:

(9) $$g\left(x\right)=\sum_{i=1}^{N_{G}}M_{1}^{T}\left(x\right)G_{1i}M_{2}^{T}\left(y\right)G_{2i}M_{3}^{T}\left(z\right)G_{3i}\cdots =\sum_{i=1}^{N_{G}}\prod_{j=1}^{N_{D}}M_{j}^{T}G_{ji}$$

where $N_{D}$ is the number of dimensions of the problem space, $\Omega \in R^{N_{D}}$. The functions $M_{j}\left(x_{j}\right)$, $j=1,2,\ldots ,N_{D}$ are the vectors of approximation functions in term of the *j*-th coordinate direction, $\left(x,y,z,p_{1},p_{2},\ldots \right)$, and vectors $G_{ji}$ are coefficient vectors associated with the *j*-th coordinate for the *i*-th mode.

The Lagrange interpolation functions or any other approximation functions could be used to define the approximation functions $M_{j}\left(x_{j}\right)$. It was also possible to define a different order of approximations in different coordinate directions to capture specific physical characteristics.

As stated before, the generic function g(x) might be a known or an unknown field function. In the former case, the coefficients vectors $G_{ji}$ were considered known and could be obtained using an iterative process given below in Section 4. On the other hand, if generic function g(x) was an unknown field function (e.g., displacement fields), the coefficient vectors $G_{ji}$ were unknown and had to be obtained using the governing equations and PGD method as described later in Section 5.

## 4. SAR of a Given Field Function

Consider g(x) as a given (known) field function in the problem space, $\Omega \in R^{N_{D}}$. We were interested in obtaining a SAR of g(x) in terms of a set of univariate approximation functions, $M_{j}$, and a set of coefficients, $G_{ji}$, as given in Equation (9). Regarding $M_{j}$, different sets of approximation functions could be selected generally without any specific restrictions. However, the coefficient vectors $G_{ji}$ should be computed as explained here. Without the loss of generality, consider that we already had the first (n−1) terms (modes) of the summation in Equation (9), and we wanted to find the next term (mode), *n*. Then, the residual of the first (n−1) modes was as follows:

(10) $$r^{n-1}\left(x\right)=g\left(x\right)-\sum_{i=1}^{n-1}\prod_{j=1}^{N_{D}}M_{j}^{T}G_{ji}$$

Therefore, the *n*-th mode had to be obtained in such a way that determined an approximation for the residual $r^{n-1}\left(x\right)$. We had:

(11) $$\prod_{j=1}^{N_{D}}M_{j}^{T}G_{jn}\approx r^{n-1}\left(x\right)$$

To find the coefficients $G_{jn}$ that gave the best approximation for the residual $r^{n-1}\left(x\right)$, the following error norm had to be minimized:

(12) $$∥e∥=\int_{\Omega }{\left(\prod_{j=1}^{N_{D}}M_{j}^{T}G_{jn}-r^{n-1}\left(x\right)\right)}^{2}d\Omega$$

In other words, the coefficients $G_{jn}$ had to be obtained in a least square sense. To minimize the error norm ∥e∥, its first derivatives with respect to all elements of the coefficient vectors $G_{jn},j=1,...,N_{D}$ had to vanish. Therefore, we had the following set of equations:

(13) $$\frac{\partial ∥e∥}{\partial G_{dn}^{\alpha }}=0,\;\;\;d=1,2,...,N_{D},\;\;\;\alpha =1,2,...,N_{d}$$

where $G_{dn}^{\alpha }$ is the α-th element of the coefficient vector $G_{dn}$ in the *d*-th direction of the coordinate space. $N_{d}$ is the number of approximation functions in the *d*-th direction. The equations given in Equation (13) could be arranged as follows:

(14) $$\begin{array}{c} \frac{\partial ∥e∥}{\partial G_{dn}^{\alpha }}=0\Rightarrow \int_{\Omega }M_{d}^{\alpha }\left(M_{d}^{T}G_{dn}\right)\prod_{\begin{array}{c} j=1\\ j\neq d \end{array}}^{N_{D}}{\left(M_{j}^{T}G_{jn}\right)}^{2}d\Omega =\\ \int_{\Omega }M_{d}^{\alpha }\prod_{\begin{array}{c} j=1\\ j\neq d \end{array}}^{N_{D}}\left(M_{j}^{T}G_{jn}\right)r^{n-1}\left(x\right)d\Omega \end{array}$$

where $M_{d}^{\alpha }$ is the α-th approximation function in the *d*-th direction of the coordinate space. The integrals in Equation (14) could be separated and the equation could be arranged as follows:

(15) $${\left(\int M_{d}^{\alpha }M_{d}dx_{d}\right)}^{T}G_{dn}=\frac{\int M_{d}^{\alpha }dx_{d}\prod_{\begin{array}{c} j=1\\ j\neq d \end{array}}^{N_{D}}\int M_{j}^{T}G_{jn}dx_{j}}{\prod_{\begin{array}{c} j=1\\ j\neq d \end{array}}^{N_{D}}\int {\left(M_{j}^{T}G_{jn}\right)}^{2}dx_{j}}$$

Consider that $d=1,2,...,N_{D}$ and $\alpha =1,2,...,N_{d}$; then, Equation (15) would consist of $N_{D}$ systems of equations, each one consisting of $N_{d}$ algebraic nonlinear equations.

The above procedure is the basic mathematical description to obtain the SAR of a given generic field function g(x) in terms of an arbitrary set of approximation functions $M_{j}\left(x_{j}\right)$. This procedure would be simplified considerably if the linear Lagrange interpolation function was selected as the approximation function. Finally, the simplified system of equations given in Equation (15) could be solved using the fixed point iteration method to obtain all coefficient vectors $G_{jn}$. More explanations and other techniques to obtain the SAR of a known field function can be found in references [22,23].

The above procedure gave the *n*-th mode of the SAR. This process could be continued to generate next subsequent modes one by one. A convergence criterion was also needed to terminate the whole process and limit the number of modes to $N_{G}$ as appeared in Equation (9).

## 5. Proper Generalized Decomposition

As explained in Section 4, a SAR can be constructed for any given (known) functions in the problem space, Ω. For example, the elements of the elasticity matrix, $C_{ef}\left(x\right)$, are known functions in the problem space, Ω. In addition, the body force components, $f_{a}\left(x\right)$, are also given functions. The traction components $t_{a}\left(x\right)$ are known functions defined on the plate surfaces ($R^{N_{D}-1}$). It was possible to use the technique given in Section 4 to construct a SAR for each of these functions as follows:

(16) $$C_{ef}\left(x\right)=\sum_{i=1}^{N_{C_{ef}}}\prod_{j=1}^{N_{D}}M_{j}^{T}C_{efji}$$

(17) $$f_{a}\left(x\right)=\sum_{i=1}^{N_{f_{a}}}\prod_{j=1}^{N_{D}}M_{j}^{T}F_{aji}$$

(18) $$t_{a}\left(x\right)=\sum_{i=1}^{N_{t_{a}}}\prod_{j=1}^{N_{D}-1}M_{j}^{T}T_{aji}$$

where, $N_{C_{ef}}$, $N_{f_{a}}$ and $N_{t_{a}}$ are the numbers of modes in the SAR of $C_{ef}$, $f_{a}$ and $t_{a}$, respectively. The vectors $C_{efji}$, $F_{aji}$ and $T_{aji}$ are corresponding vectors of coefficients regarding the *i*-th mode in the *j*-th space direction.

The PGD technique is an approach to obtain a SAR for unknown field functions. For instance, the displacement components $u_{x}\left(x\right)$, $u_{y}\left(x\right)$ and $u_{z}\left(x\right)$ are unknown field functions which must be obtained using PGD based on the weak form of governing equations given in Equations (4)–(6).

Consider displacement field $u_{a}\left(x\right)$, where a∈x,y,z, without the loss of generality, assume that the first (n−1) terms (modes) of their separated representation are known, and we wanted to find the next mode *n* as follows:

(19) $$u_{a}=\sum_{i=1}^{n-1}\prod_{j=1}^{N_{D}}M_{j}^{T}u_{aji}+\prod_{j=1}^{N_{D}}M_{j}^{T}u_{ajn}$$

The first variation of the displacement component $u_{a}^{*}$ could be obtained as follows:

(20) $$u_{a}^{*}=\sum_{d=1}^{N_{D}}M_{d}\prod_{\begin{array}{c} j=1\\ j\neq d \end{array}}^{N_{D}}M_{j}^{T}u_{ajn}$$

In other words, the unknown coefficient vectors $u_{ajn}$ had to be obtained in such a way that resulting displacement fields $u_{a}$ as given in Equation (19) satisfy the weak form of the governing equations. This is a sequential approach and, after obtaining the *n*-th mode, the process repeats to obtain subsequent modes. A termination criterion should stop the process after reaching the desired accuracy level.

Now, Equations (16)–(20) were introduced into the weak form given in Equation (7). This gave a set of nonlinear algebraic equations that had to be solved to obtain the coefficient vectors $u_{ajn}$. The simplest approach for solving this system of equations is the fixed point iteration method. In each iteration, just one vector is considered as unknown and the other ones are considered known. This process continues until reaching a termination criteria for the fixed point iterations. In summary, the PGD technique consists of two loops. The outer loop try to enrich the solution by adding more modes, and the inner loop runs on the fixed point iteration to solve the system of nonlinear algebraic equations.

The above procedure is the standard PGD technique and there are a lot of detailed explanations regarding theoretical formulations and, also, the practical implementation of the technique. For instance, refer to [22].

## 6. Numerical Examples

Three examples were presented in this section considering parametric study. The first two examples were adopted in such a way that the results for some specific values of material parameters could be validated using the exact solutions available in the literature. The last example provided further studies on the parametric solution of tri-directional FGM plates. The resulting parametric study was then used to perform a material distribution design (optimization) based on a failure criteria.

### 6.1. Example 1

**Figure 1:**
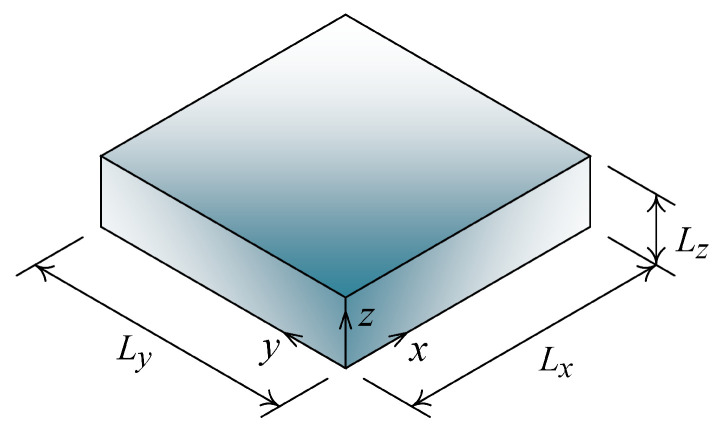
Schematic representation of plate, coordinate system and their dimensions.

In the first example, the parametric study of the bending behavior of an orthotropic FGM plate based on one material parameter was considered and the results were compared with the exact closed-form solution presented in [24] for some specific values of the material parameter. The plate and the coordinate system are schematically shown in Figure 1. The plate dimensions were given as $L_{x}=L_{y}=3$ m and $L_{z}=1$ m. Three sets of boundary conditions, SSSS, CCCC and CSFF, were considered and explicitly defined in Table 2. A distributed sinusoidal traction was applied over the top surface of the plate ($z=L_{z}$):

(21) $$t_{z}=q_{max}\sin\frac{\pi x}{L_{x}}\sin\frac{\pi y}{L_{y}}$$

where $t_{z}$ is the vertical component of the traction vector on the top surface. Other components of the traction vectors on all parts of the natural boundaries were zero. The FGM mechanical characteristics of the plate changed exponentially along the thickness (in the *z*) direction and the resulting elasticity matrix C was given as follows [24]:

(22) $$C=C^{0}e^{pz}$$

where *p* is the exponential factor that determines the material distribution in the *z* direction. The three cases of material parameter, p=0, p<0 and p>0, corresponded to homogeneous, graded soft and graded stiff materials, respectively. In Equation (22), the base elasticity matrix $C^{0}$ was obtained using the engineering material constants given in Table 3 and using the generalized Hook law for orthotropic materials [24].

In the present example, a parametric study was conducted based on factor *p* in range [−1,1]. Therefore, the problem space, Ω, consisted of three geometrical coordinates, *x*, *y*, *z*, and one material parameter *p*. In other words, the elasticity problem was solved in a 4D problem space (x,y,z,p) using the PGD technique. It reduced this 4D problem into a set of 1D sub-problems, each of them in a single dimension of the problem space. Therefore, this technique reduced the computational cost significantly, and made it possible to solve problems in high-dimensional spaces.

**Figure 2:**
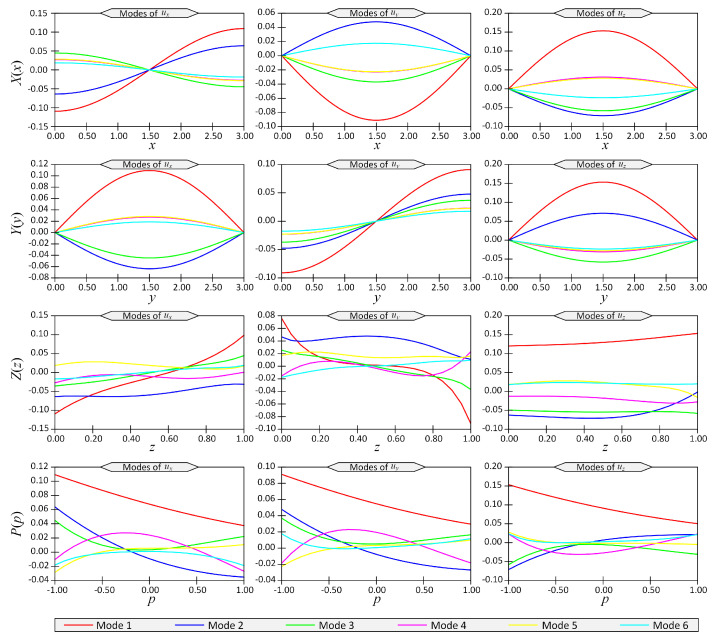
The first six modes of displacement components ($u_{x},u_{y},u_{z}$) with respect to space coordinates (x,y,z,p) for Example 1 for boundary conditions case SSSS.

After solving this 4D problem, the separated representation of displacement fields was obtained. The first six modes of displacement components $u_{x}$, $u_{y}$ and $u_{z}$ versus space coordinates *x*, *y*, *z* and *p* are shown in Figure 2 for the boundary conditions SSSS. Note that the vertical axes in this figure show 1D functions X(x), Y(y), Z(z) and P(p) (see Equation (8)). These functions did not have a clear physical description or unit, but their multiplications represented physical quantities (here, displacements) with a unit of length.

To validate the results, three specific material parameters p=−1, p=0 and p=1 were considered, and the resulting values of displacements $u_{x}$, $u_{y}$ and $u_{z}$ were obtained at two locations (x,y,z)=(0.75,0.75,0) and (0.75,0.75,1), which were located at the bottom and top plate faces, respectively. The displacements are presented in Table 4. To study the effect of grid sizes, three different combinations of 1D grid sizes in each space directions were selected. The number of nodes in each direction was arranged as $\left\{n_{1},n_{2},n_{3},n_{4}\right\}$ and shown at the top of each column in Table 4. In addition, in this table, the closed-form solutions given in [24] were compared with the present results. The percentage error, with respect to these reference solutions, was shown in parenthesis next to each displacement value. The results showed an excellent agreement between PGD and reference solutions, even for course grid sizes.

**Figure 3:**
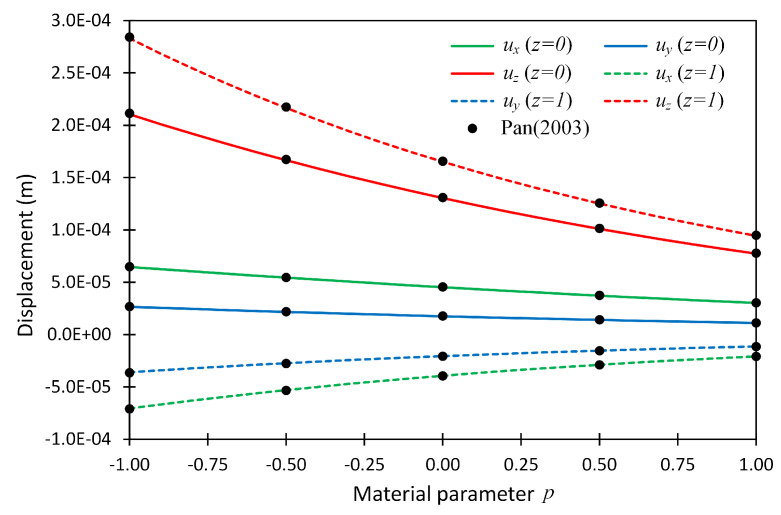
The displacement components at two locations (x,y,z) = (0.75,0.75,0) and (0.75,0.75,1) versus material parameter *p* for Example 1 considering boundary conditions SSSS; the reference solution given in (Pan 2003) [24] is shown by solid dots.

The displacement values at two above-mentioned locations were also plotted versus material parameter *p*, and shown in Figure 3 for boundary condition case SSSS. In this figure, the line graphs show the present solution, while the solid dots show the reference solutions given in [24].

**Figure 4:**
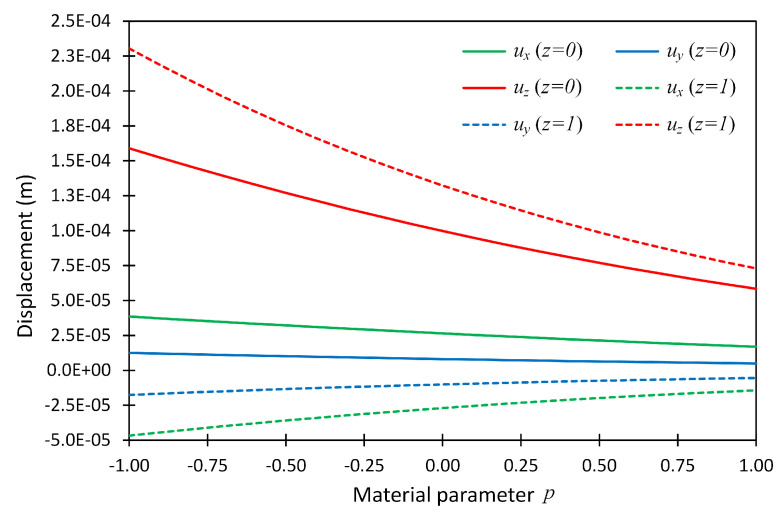
The displacement components at two locations (x,y,z)=(0.75,0.75,0) and (0.75,0.75,1) versus material parameter *p* for Example 1 considering boundary conditions CCCC.

**Figure 5:**
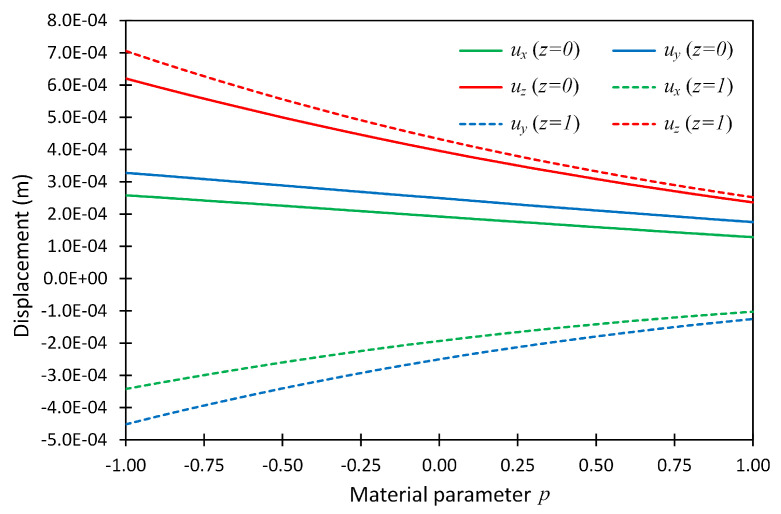
The displacement components at two locations (x,y,z)=(0.75,0.75,0) and (0.75,0.75,1) versus material parameter *p* for Example 1 considering boundary conditions CSFF.

The displacements $u_{x}$, $u_{y}$ and $u_{z}$ for boundary conditions CCCC and CSFF were also calculated in a similar manner and were plotted for the full range of material parameter *p* at the above-mentioned points, and are shown in Figure 4 and Figure 5. This example revealed that the PGD technique made it possible to obtain the system performance for the full range of a material parameter instead of considering some specific values.

To obtain a simple estimate for the computational advantages of the PGD technique versus traditional mesh-based methods (e.g., FEM), it should be mentioned that for the coarsest grid in the present example, we had {21×21×11×11} nodal points (see Table 4). In the traditional mesh-based methods, such a grid would lead to 53361 nodes and its computational complexity would be of the order of $O(53361^{2})$, whereas, in the PGD technique, the computational complexity would be of the order of $O\left(21^{2}\right)+O\left(21^{2}\right)+O\left(11^{2}\right)+O\left(11^{2}\right)$. This considerable reduction in computational costs was a direct result of the reduced order modeling that reduced a 4D problem into a set of 1D problems.

### 6.2. Example 2

For the second validation example, the benchmark problem presented in [19] was considered. In this problem, a unidirectionally reinforced high-modulus graphite epoxy composite FGM plate of size $L_{x}\times L_{y}\times L_{z}$, as shown in Figure 1, was considered. Two cases of fiber orientation were studied. In the first case, the reinforcements were placed along the *x* axis (i.e., $0^{\circ }$), and in the second one, were placed along the *y* axis (i.e., $90^{\circ }$). The volume fraction of the fibers, $V^{f}$, was considered varying with respect to the *x* axis, with symmetry about the mid span of the plate, as follows:

(23) $$V^{f}=V_{min}^{f}+\left(V_{max}^{f}-V_{min}^{f}\right)w\left(p\right)$$

(24) $$w\left(p\right)=4\left(2p-1\right){\left(\frac{x}{L_{x}}\right)}^{2}-4\left(2p-1\right)\left(\frac{x}{L_{x}}\right)+p$$

where $V_{min}^{f}$ and $V_{max}^{f}$ are the minimum and maximum values of the fiber volume fraction, respectively. The values $V_{min}^{f}=0.1$ and $V_{max}^{f}=0.7$ were assumed from the practical point of view. The weighting function w(p) determined the distribution of the fiber volume fraction across the plate in terms of the material parameter p∈[0,1], which controlled the distribution. The distribution given in Equations (23) and (24) was a generalization of a simpler form that has been considered in other works such as [19,25,26,27].

**Figure 6:**
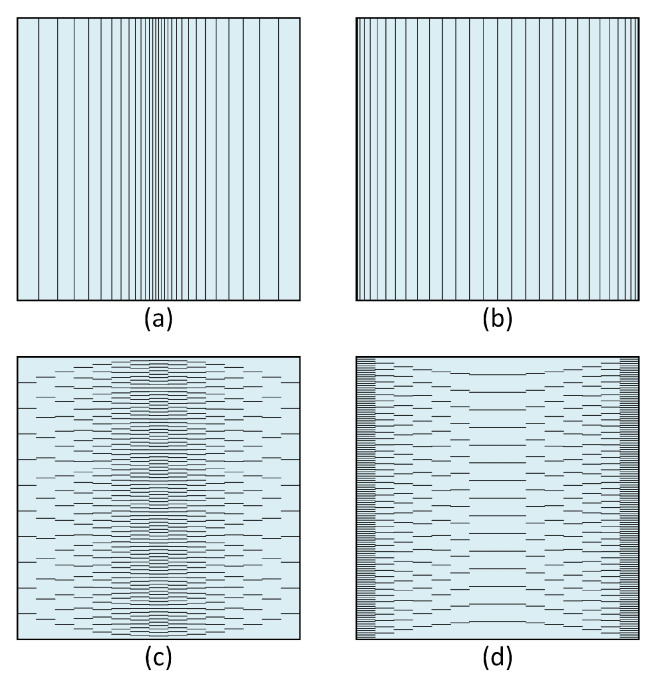
Schematic representation of fiber orientations and volume fraction distributions for the FGM composite plate considered in Example 2; (**a**) $90^{\circ }$, p=0; (**b**) $90^{\circ }$, p=1; (**c**) $0^{\circ }$, p=0; (**d**) $0^{\circ }$, p=1.

The two cases of fiber orientations, i.e., $0^{\circ }$ and $90^{\circ }$, are schematically shown in Figure 6 for two specific cases of material parameters p=0 and p=1. As was visible in this figure, the fiber volume fraction at the plate mid-span was greater than either sides for the extreme case p=0. This condition was reversed for another extreme case p=1.

The engineering elastic constants of the components of the unidirectional high-modulus graphite epoxy composite are given in Table 5. For the fiber phase, the subscripts *L* and *T* stand for the fiber longitudinal and transverse directions, respectively. For the matrix phase, an isotropic behavior was considered.

To obtain the macroscopic engineering elastic constants of the composite materials, there were several micromechanics relations given in the literature. In the present example, we used the Halpin–Tsai relation, which has been widely accepted as sufficiently accurate and simple to use. The Halpin–Tsai relation was given below, and more details about this micromechanical model can be found in references such as [28].

(25) $$\begin{array}{cc} \; & E_{L}=E_{L}^{f}V^{f}+E^{m}V^{m}\\ \; & E_{T}=E^{m}\frac{1+2\lambda V^{f}}{1-\lambda V^{f}}\;\;\;\mathrm{where}\;\;\;\lambda =\frac{E_{T}^{f}-E^{m}}{E_{T}^{f}+2E^{m}}\\ \; & G_{LT}=G^{m}\frac{1+\lambda V^{f}}{1-\lambda V^{f}}\;\;\;\mathrm{where}\;\;\;\lambda =\frac{G_{LT}^{f}-G^{m}}{G_{LT}^{f}+G^{m}}\\ \; & G_{TT}=G^{m}\frac{1+\lambda V^{f}}{1-\lambda V^{f}}\;\;\;\mathrm{where}\;\;\;\lambda =\frac{G_{TT}^{f}-G^{m}}{G_{TT}^{f}+G^{m}}\\ \; & \nu_{LT}=\nu_{LT}^{f}V^{f}+\nu^{m}V^{m}\\ \; & \nu_{TL}=\nu_{LT}\frac{E_{T}}{E_{L}}\\ \; & \nu_{TT}=\frac{E_{T}}{2G_{TT}}-1 \end{array}$$

where the superscripts *f* and *m* show that those engineering constants referred to the fiber phase and matrix phase, respectively.

After obtaining the engineering constants using the Halpin–Tsai model as given in Equation (25) and volume fraction given in Equation (23), the elements of the elasticity matrix $C_{ef}\left(x\right)$ could be obtain as regular for orthotropic materials (e.g., refer to [21]). Note that, for each case of fiber orientations, $0^{\circ }$ and $90^{\circ }$, the L and T directions in Equation (25) should be aliened in proper *x* and *y* directions.

The loading of the plate was similar to the previous example, and a sinusoidal traction in the *z* direction was distributed over the top surface of the plate. Three cases of boundary conditions, SSSS, CCCC and CSFF, were applied on all boundaries similar to the previous example, and its details are given in Table 2. The problem was solved for the square plate, $L_{x}=L_{y}$, considering a different plate thickness, $L_{z}$. To present the displacements, the following relation was used to make them dimensionless:

(26) $${\bar{u}}_{z}=\left(\frac{100E^{m}}{q_{max}L_{z}S^{4}}\right)u_{z}$$

where $S=L_{x}/L_{z}$ is the thickness ratio and $q_{max}$ is the maximum load intensity (see Equation (21)).

To validate the results, the dimensionless deflection ${\bar{u}}_{z}$ was computed at the center point of the plate at location $\left(L_{x}/2,L_{y}/2,L_{z}/2\right)$ for boundary conditions SSSS and material parameter p=0. These values are given in Table 6. To study the effect of grid sizes, four different combinations of 1D grid sizes in each space direction were selected and shown at the top of each column in Table 6. In addition, in this table, the closed-form solutions given in [19] were compared with the present values. The percentage error with respect to the reference solution was shown in parenthesis next to each values. The results showed an excellent agreement between PGD results and reference solutions.

**Figure 7:**
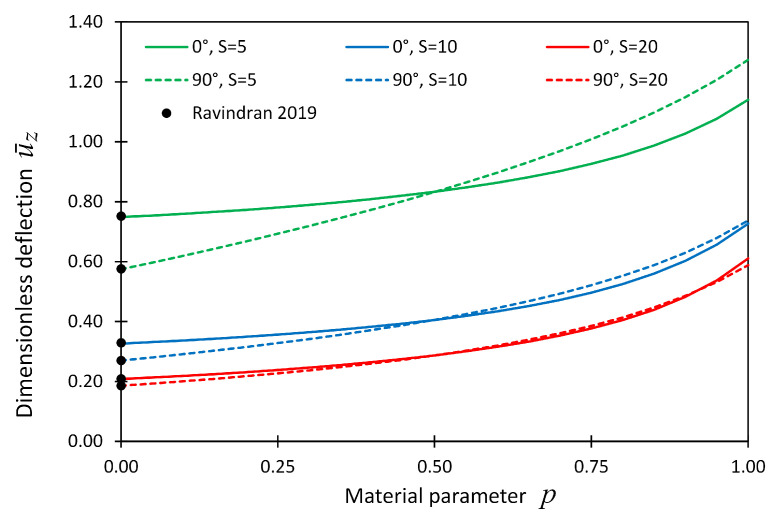
The dimensionless displacement, ${\bar{u}}_{z}$, at locations (x,y,z) = ($L_{x}/2,L_{y}/2,L_{z}/2$) versus material parameter *p* for Example 2 considering boundary conditions SSSS; the reference solution given in (Ravindran 2019) [19] is shown by solid dots.

The dimensionless deflection, ${\bar{u}}_{z}$, at the plate center point was also plotted versus material parameter *p* and shown in Figure 7 for boundary condition cases SSSS. In this figure, the line graphs show the present solution, while the solid dots show the reference solutions given in [19].

**Figure 8:**
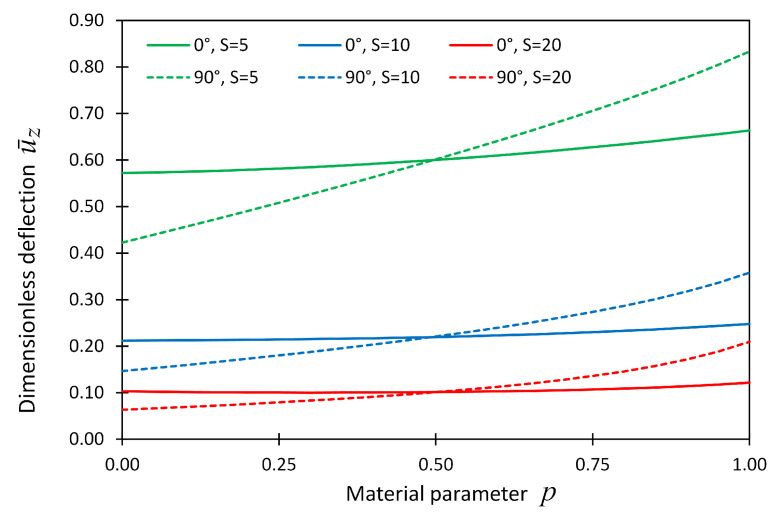
The dimensionless displacement, ${\bar{u}}_{z}$, at locations (x,y,z) = ($L_{x}/2,L_{y}/2,L_{z}/2$) versus material parameter *p* for Example 2 considering boundary conditions CCCC.

**Figure 9:**
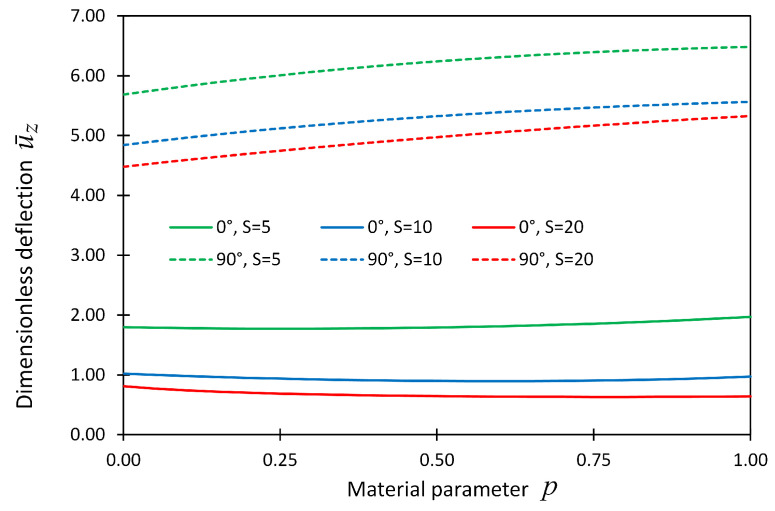
The dimensionless displacement, ${\bar{u}}_{z}$, components at locations (x,y,z) = ($L_{x}/2,L_{y}/2,L_{z}/2$) versus material parameter *p* for Example 2 considering boundary conditions CSFF.

Figure 8 and Figure 9 also show the dimensionless displacement ${\bar{u}}_{z}$ with respect to material parameter *p* for boundary condition cases CCCC and CSFF, respectively.

### 6.3. Example 3

**Figure 10:**
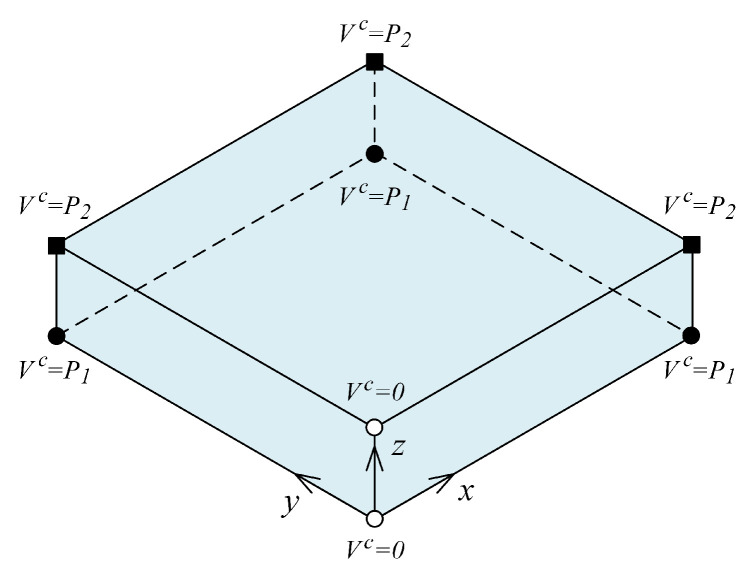
Material parameters for Example 3.

In this example, the parametric study and material distribution design (optimization) of a tri-directional ceramic–metal FGM thick plate were considered. The parametric study made it possible to use any optimization techniques to find the best material distribution based on a failure theory. The plate dimensions and coordinate system are shown in Figure 1 considering $L_{x}=L_{y}=3$ and $L_{z}=1$. Here, two material parameters were considered to parameterize the material distribution in all spacial directions *x*, *y* and *z*. Figure 10 shows that the material parameters $p_{1}\in \left[0,1\right]$ and $p_{2}\in \left[0,1\right]$ gave the ceramic volume fraction $V^{c}$ at six corners of the plate. The remaining two corners were maintained at $V^{c}=0$. A tri-linear distribution was considered for $V^{c}\left(x,y,z\right)$ for internal points of the plate as given below.

(27) $$V^{c}\left(x,y,z\right)=w_{1}\left(x,y,z\right)p_{1}+w_{2}\left(x,y,z\right)p_{2}$$

(28) $$w_{1}\left(x,y,z\right)=\left(1-z\right)\left(x+y-xy\right)$$

(29) $$w_{2}\left(x,y,z\right)=z\left(x+y-xy\right)$$

where, $w_{1}$ and $w_{2}$ are distribution functions. The material parameters combination $p_{1}=p_{2}=0$ corresponds to the homogeneous metallic phase, whereas any other combinations of $p_{1}$ and $p_{2}$ correspond to a gradual change of material characteristics in the *x*, *y* and *z* directions.

The engineering constants for ceramic and metal phases are given in Table 7. The mixture rule was used to obtain the engineering elastic constants (Young modules and Poisson ratio) at each point of the plate using the above-explained ceramic volume fraction distribution. After that, the elements of elasticity tensor $C_{ef}$ could be obtained using the resulting engineering constants. In addition, the tensile and compressive allowable stresses are also given in Table 7 for constitutive materials. The mixture rule was also used to obtain the resulting allowable tensile and compressive stresses at each point.

A uniform traction $t_{z}=10^{7}$ was applied at the top surface, while a traction-free condition was considered for the other natural boundaries. The essential boundary condition CFSF was considered here, and its details are given in Table 2.

The parametric study was performed here based on two material parameters $p_{1}$ and $p_{2}$. Therefore, the problem space consisted of three geometrical coordinates and two material parameters. In other words, the elasticity problem was solved in a 5D problem space $\left(x,y,z,p_{1},p_{2}\right)$ using the PGD technique by reducing it into a set of 1D sub-problems, each of them in a single direction of the problem space.

**Figure 11:**
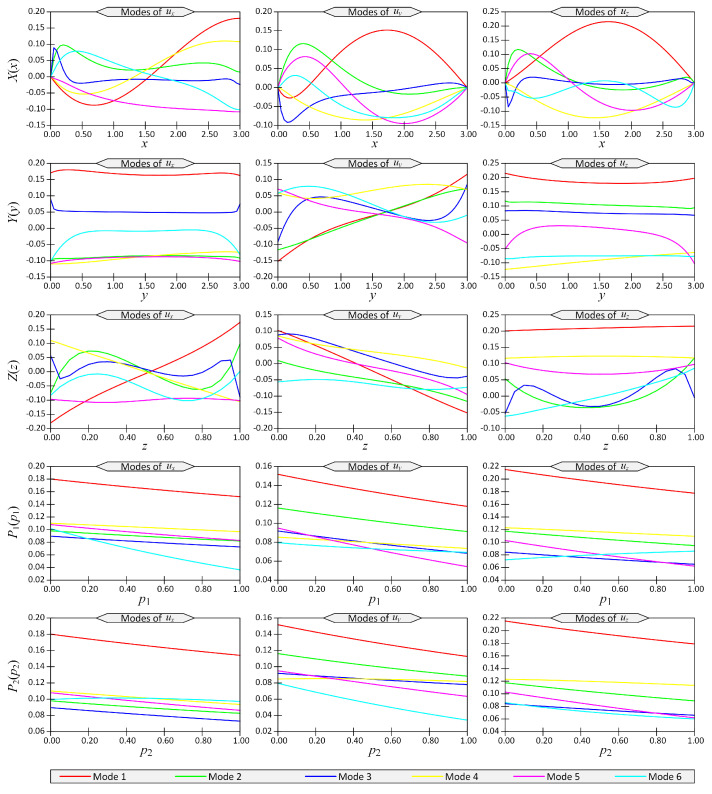
The first six modes of displacement components ($u_{x},u_{y},u_{z}$) with respect to space coordinates ($x,y,z,p_{1},p_{2}$) for Example 3.

Using the PGD technique, the separated representation of the displacements was obtained. The first six modes of displacements $u_{x}$, $u_{y}$ and $u_{z}$ versus coordinates *x*, *y*, *z*, $p_{1}$ and $p_{2}$ are shown in Figure 11 for the above-mentioned loading and boundary conditions. The vertical axes in this figure show the 1D functions X(x), Y(y), Z(z), $P_{1}\left(p_{1}\right)$ and $P_{2}\left(p_{2}\right)$ (see Equation (8)).

Now, all displacement components were available and it was possible to calculate the strain and stress fields using Equations (3) and (2), respectively.

After calculating the stress components and, also, the allowable stresses at each point of the problem space Ω, a failure criteria could be used to evaluate the state of the material at each point. To perform this, here, the Coulomb–Mohr static failure criteria, which is commonly used for materials with different compressive and tensile behaviors, was used to obtain the factor of safety due to failure at each point. The Coulomb–Mohr factor of safety was defined as below.

(30) $$\eta \left(x,y,z,p_{1},p_{2}\right)=\frac{S_{cmp}}{\frac{m-1}{2}\left(\sigma_{1}+\sigma_{2}+\sigma_{3}\right)+\frac{m+1}{2}\sqrt{\frac{1}{2}({\left(\sigma_{1}-\sigma_{2}\right)}^{2}+{\left(\sigma_{2}-\sigma_{3}\right)}^{2}+{\left(\sigma_{3}-\sigma_{1}\right)}^{2})}}$$

where $m=S_{cmp}/S_{tns}$. $S_{ten}$ and $S_{cmp}$ are allowable tensile and compressive stresses for any point of FGM plate based on the material properties given in Table 7 and volume fraction given in Equation (27). $\sigma_{1}$, $\sigma_{2}$ and $\sigma_{3}$ are principal stresses.

For each material parameter combination ($p_{1},p_{2}$), it was possible to find the minimum value of the factor of safety as follows:

(31) $$\eta_{min}\left(p_{1},p_{2}\right)=\min{\left(\eta (x,y,z,p_{1},p_{2})\right)}_{x,y,z}$$

**Figure 12:**
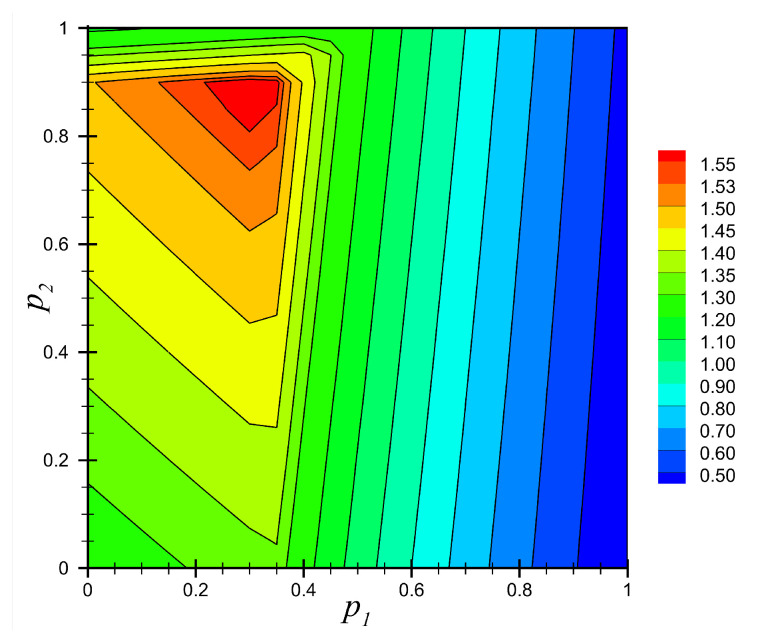
Contour plot of the field of minimum factor of safety, $\eta_{min}\left(p_{1},p_{2}\right)$, for the full range of material parameters $p_{1}$ and $p_{2}$ for Example 3.

Figure 12 shows the contour plot of the distribution of the minimum factor of safety $\eta_{min}$ over the full range of material parameters $p_{1}\in \left[0,1\right]$ and $p_{2}\in \left[0,1\right]$. This figure shows that $\eta_{min}$ had a maximum value of $\eta_{min}=1.57$ near the location $\left(p_{1},p_{2}\right)=\left(0.3,0.9\right)$. In other words, this combination of material parameters showed the optimum material distribution regarding the selected failure criteria and the material parameterization.

**Figure 13:**
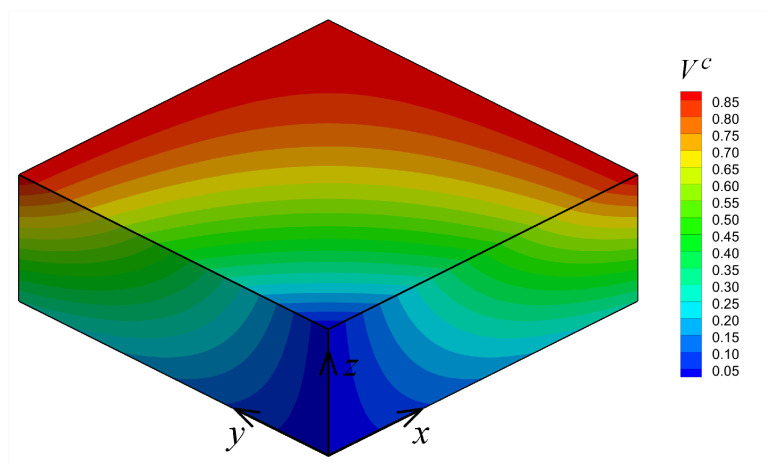
Contour plot of volume fraction distribution $V^{c}\left(x,y,z\right)$ for the optimum material parameters $\left(p_{1},p_{2}\right)=\left(0.3,0.9\right)$ for Example 3.

**Figure 14:**
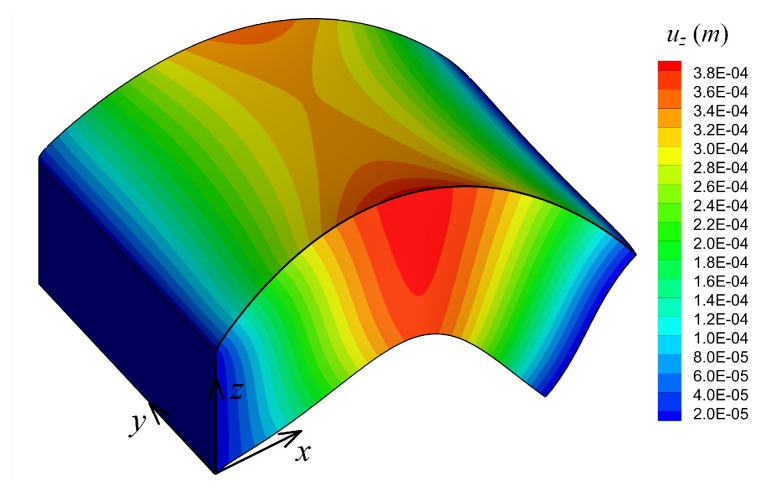
Deformed configuration (not scaled) and contour plot of displacement $u_{z}$ for the optimum material parameters $\left(p_{1},p_{2}\right)=\left(0.3,0.9\right)$ for Example 3.

Figure 13 shows the contour plot of the volume fraction, $V^{c}$, for the above-mentioned optimum material distribution. The plate deformed configuration (with exaggerated displacements) and contours of displacement field $u_{z}$ are shown in Figure 14 for the case of the optimum material distribution.

## 7. Conclusions

PGD makes advanced parametric analyses possible, by considering parameters as model extra-coordinates, while circumventing the resulting curse of dimensionality by using separated representations. When applying a separated representation of the field functions, the solution of a high-dimensional boundary value problem was reduced to a sequence of low dimensional (1D) sub-problems. This reduced computational costs significantly and allowed to deal with high-dimensional engineering problems in a reasonable runtime on office computers. This technique would be especially very helpful in the case of thick FGM plate bending problems, in which the material distribution design/optimization is a big challenge. In this context, plate bending theories suffered from the lack of accuracy in considering shear effects and using the 3D theory of elasticity was computationally expensive. In the present work, PGD was applied, for the first time, in a parametric study and material distribution design/optimization of FGM thick plates considering any types of loading and boundary conditions.

## Figures and Tables

**Table 1:** The indices sets {S} used in Equation (7).

Equation	Term	a	b	c	d	e	f
Equation (4)	1	x	x	x	y	1	1
	2	x	x	y	y	1	2
	3	x	x	z	z	1	3
	4	x	y	y	x	4	4
	5	x	y	x	y	4	4
	6	x	z	x	z	6	6
	7	x	z	z	x	6	6
Equation (5)	1	y	x	y	x	4	4
	2	y	x	x	y	4	4
	3	y	y	x	x	1	2
	4	y	y	y	y	2	2
	5	y	y	z	z	2	3
	6	y	z	z	y	5	5
	7	y	z	y	z	5	5
Equation (6)	1	z	x	x	z	6	6
	2	z	x	z	x	6	6
	3	z	y	z	y	5	5
	4	z	y	y	z	5	5
	5	z	z	x	x	1	3
	6	z	z	y	y	2	3
	7	z	z	z	z	3	3

**Table 2:** Explicit definition of plate boundary conditions regarding to Examples 1, 2 and 3.

B.C. Type	Face (x=0)	Face ($x=L_{x}$)	Face (y=0)	Face ($y=L_{y}$)
SSSS	$u_{y}=u_{z}=0$	$u_{y}=u_{z}=0$	$u_{x}=u_{z}=0$	$u_{x}=u_{z}=0$
CCCC	$u_{x}=u_{y}=u_{z}=0$	$u_{x}=u_{y}=u_{z}=0$	$u_{x}=u_{y}=u_{z}=0$	$u_{x}=u_{y}=u_{z}=0$
CSFF	$u_{x}=u_{y}=u_{z}=0$	—	$u_{y}=u_{z}=0$	—
CFSF	$u_{x}=u_{y}=u_{z}=0$	$u_{y}=u_{z}=0$	—	—

**Table 3:** Base engineering elastic constants for Example 1.

$E_{x}$	6.89476	GPa
$E_{y}$	172.369	GPa
$E_{z}$	6.89476	GPa
$G_{xy}$	3.44738	GPa
$G_{yz}$	3.44738	GPa
$G_{zx}$	1.37895	GPa
$\nu_{xy}$	0.01	
$\nu_{yz}$	0.25	
$\nu_{zx}$	0.25	

**Table 4:** The displacement components for three values of material parameter *p*, for Example 1, for boundary conditions SSSS obtained using different grid sizes.

Dis.	Loc.	*p*	Ref. [24]	Present		
				{21 × 21 × 11 × 11}	{41 × 41 × 15 × 15}	{61 × 61 × 21 × 21}
$u_{x}$	z = 0	−1	6.4876	6.3892 (1.5)	6.4416 (0.7)	6.4629 (0.4)
		0	4.5491	4.4825 (1.5)	4.5182 (0.7)	4.5324 (0.4)
		1	3.0359	2.9864 (1.6)	3.0128 (0.8)	3.0236 (0.4)
	z = 1	−1	−7.0921	−6.9544 (1.9)	−7.0267 (0.9)	−7.0577 (0.5)
		0	−3.9492	−3.8791 (1.8)	−3.9161 (0.8)	−3.9317 (0.4)
		1	−2.0888	−2.0502 (1.9)	−2.0705 (0.9)	−2.0792 (0.5)
$u_{y}$	z = 0	−1	2.6853	2.5854 (3.7)	2.6357 (1.8)	2.6599 (0.9)
		0	1.7737	1.7070 (3.8)	1.7406 (1.9)	1.7568 (1.0)
		1	1.1232	1.0779 (4.0)	1.1007 (2.0)	1.1117 (1.0)
	z = 1	−1	−3.643	−3.4934 (4.1)	−3.5678 (2.1)	−3.6046 (1.1)
		0	−2.0733	−1.9945 (3.8)	−2.0338 (1.9)	−2.0531 (1.0)
		1	−1.1419	−1.0992 (3.7)	−1.1206 (1.9)	−1.1310 (1.0)
$u_{z}$	z = 0	−1	21.134	20.823 (1.5)	20.987 (0.7)	21.055 (0.4)
		0	13.095	12.918 (1.4)	13.012 (0.6)	13.050 (0.3)
		1	7.7749	7.6602 (1.5)	7.7207 (0.7)	7.7457 (0.4)
	z = 1	−1	28.412	28.076 (1.2)	28.249 (0.6)	28.322 (0.3)
		0	16.568	16.384 (1.1)	16.480 (0.5)	16.519(0.3)
		1	9.4808	9.3593 (1.3)	9.4229 (0.6)	9.4492 (0.3)

The numbers in parenthesis show the percentage error with respect to the reference solution. The displacements are given on bottom and top surfaces at location x=y=0.75 m. All displacement values are given in $10^{-5}$.

**Table 5:** Engineering elastic constants of constitutive materials for Example 2.

Fiber	$E_{L}^{f}$	388	GPa
	$E_{T}^{f}$	7.17	GPa
	$G_{LT}^{f}$	6.79	GPa
	$G_{TT}^{f}$	2.41	GPa
	$\nu_{LT}^{f}$	0.230	
	$\nu_{TT}^{f}$	0.486	
Matrix	$E^{m}$	3.5	GPa
	$G^{m}$	1.3	GPa
	$\nu^{m}$	0.35	

**Table 6:** The vertical dimensionless displacement, ${\bar{u}}_{z}$, for material parameter p=0, for Example 2, for different thickness ratios, *S*, obtained using different grid sizes.

θ	S	Ref. [19]	Present			
			{21 × 21 × 11 × 11}	{41 × 41 × 11 × 11}	{61 × 61 × 11 × 11}	{61 × 61 × 21 × 21}
$0^{\circ }$	5	0.752	0.742 (1.3)	0.746 (0.8)	0.746 (0.7)	0.750 (0.3)
	10	0.329	0.324 (1.5)	0.327 (0.7)	0.327 (0.6)	0.328 (0.3)
	20	0.209	0.203 (2.7)	0.207 (0.8)	0.208 (0.5)	0.208 (0.3)
$90^{\circ }$	5	0.576	0.570 (1.0)	0.572 (0.7)	0.572 (0.7)	0.575 (0.2)
	10	0.27	0.267 (0.9)	0.269 (0.5)	0.269 (0.4)	0.270 (0.1)
	20	0.186	0.183 (1.6)	0.185 (0.3)	0.186 (0.1)	0.186 (0.0)

The numbers in parenthesis show the percentage error with respect to the reference solution.

**Table 7:** Engineering material constants for Example 3.

Ceramic	$E^{c}$	348.43	GPa
	$\nu^{c}$	0.24	
	$S_{tns}^{c}$	60	MPa
	$S_{cmp}^{c}$	344.5	MPa
Metal	$E^{m}$	201.04	GPa
	$\nu^{m}$	0.3262	
	$S_{tns}^{m}$	215	MPa
	$S_{cmp}^{m}$	215	MPa

## Data Availability

Data sharing is not applicable to this article.
